# Temporal Neuromuscular Adaptations and Proteomic Signatures Following Botulinum Neurotoxin A Injection in Spastic Hemiplegia Rats

**DOI:** 10.1002/cns.71057

**Published:** 2026-07-28

**Authors:** Mengru Zhong, Huijuan Lin, Xubo Yang, Liru Liu, Yunlan Xie, Tingting Peng, Jie Luo, Lu He, Ting Gao, Hongmei Tang, Kaishou Xu

**Affiliations:** ^1^ Department of Rehabilitation, Guangzhou Women and Children's Medical Center Guangzhou Medical University Guangzhou China; ^2^ Department of Sports and Health Guangzhou Sport University Guangzhou China

**Keywords:** botulinum neurotoxin, cerebral palsy, nerve sprouting, neuromuscular junction, spasticity

## Abstract

**Background:**

Cerebral palsy (CP) is predominantly characterized by spasticity. While botulinum neurotoxin type A (BoNT‐A) effectively reduces spasticity, its transient efficacy represents a major clinical limitation. Elucidating the mechanisms that limit its duration of action may inform strategies to prolong therapeutic benefit.

**Method:**

A spastic CP rat model was established via carotid artery ligation and hypoxia in 7‐day‐old Wistar rats. On postnatal Day 21, BoNT‐A (5 U/kg) was administered to the gastrocnemius muscle. Behavioral and molecular biological assessments were performed at 4 and 12 weeks post‐injection.

**Result:**

At 4 weeks after injection, the motor performance improved, spasticity decreased (*p <* 0.05), neuromuscular junction density increased, and neurotrophic factors IGF‐1, GAP 43, and S100 increased (all *p <* 0.05). However, these functional and molecular changes diminished by 12 weeks. Further proteomic analysis revealed a shift in pathway enrichment, from protein synthesis and vesicular transport at 4 weeks to metabolic regulation at 12 weeks. Among the differentially expressed proteins, Sar1b and Rtn1, whose expression patterns paralleled with nerve sprouting, may be key regulatory factors.

**Conclusion:**

These findings indicate that BoNT‐A facilitates NMJ recovery through temporal regulation of energy metabolism, protein synthesis, and vesicular transport. Rtn1 and Sar1b may represent candidate molecular targets for extending the therapeutic effects of BoNT‐A, although further functional validation is required.

## Graphical abstract

In spastic hemiplegia rats, BoNT‐A (5 U/kg) improved motor function and reduced spasticity at 4 weeks post‐injection, but the efficacy had declined by 12 weeks. Proteomic analysis revealed 25 DEPs altered at both time points; Rtn1 and Sar1b were linked to synaptic regeneration.

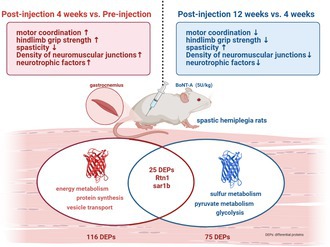

## 1 Introduction

Cerebral palsy (CP) is a group of persistent movement and postural disorders caused by non‐progressive lesions to the developing fetal or infant brain [1, 2]. The prevalence of CP ranges from 1.6 to 3.4 per 1000 live births, and its etiology is complex. Spastic CP is the most common subtype, accounting for approximately 80% of all children with CP [3, 4]. Spasticity CP commonly manifests as increased muscle tone, impaired motor control, and abnormal posture [5, 6]. Effective management of spasticity, prevention of secondary complications, and reduction of maladaptive compensatory movements and functional impairments are crucial for improving quality of life in children with CP [7].

Currently, clinical interventions for limb spasticity in CP include botulinum neurotoxin A (BoNT‐A) injections, stretching, and neurosurgical procedures [8, 9]. Among these interventions, BoNT‐A is commonly used because of its rapid onset and favorable safety profile [10, 11]. High‐quality evidence confirms that BoNT‐A safely reduces spasticity by blocking acetylcholine (ACh) release at neuromuscular junctions (NMJs), thereby inducing relaxation or temporary denervation [12, 13]. However, the effects of BoNT‐A‐induced muscle relaxation are temporary and typically last only 3–4 months in clinical practice.

Repeated injections are commonly used in clinical practice to maintain the therapeutic effects of BoNT‐A. However, repeated administration on more than three occasions may lead to increased muscle stiffness, reduced vascular density, restricted joint mobility, worsening motor impairment, and even muscle ischemic necrosis [14, 15]. Moreover, repeated injections may elicit immune responses and induce resistance to BoNT‐A, ultimately reducing therapeutic efficacy [10, 16]. Therefore, elucidating the mechanisms that limit the duration of BoNT‐A efficacy is clinically important.

Nerve sprouting after BoNT‐A injection is thought to be closely related to its limited duration of its therapeutic effects. Beginning approximately 4 days after BoNT‐A administration, local nerve terminals begin to form fine sprouts that restore ACh release and electrical activity. Subsequently, functional synapses form between the newly sprouted nerve terminals and muscle fibers, helping to restore neuromuscular transmission [17, 18]. As Ach release from the original nerve terminals recovers, the newly formed sprouts regress. By approximately 90 days after injection, neurotransmission at the original nerve terminals is largely restored. This timeline closely matches the clinical duration of BoNT‐A action [19, 20]. Thus, inhibiting nerve sprouting to prolong the effect of BoNT‐A has become an important research direction.

The process of nerve sprouting is regulated by various proteins, including neurotrophic factors, axon guidance proteins, and synaptic scaffold proteins [21, 22]. Previous studies have reported that inhibiting Agrin and IGF1R expression in healthy rats could effectively suppress nerve sprouting [16, 23]. However, clinical evidence indicates that the plasma metabolomics and proteomics profiles in children with CP differ significantly from those of healthy controls, suggesting that molecular mechanisms identified in healthy animals may not be directly applicable to CP [24]. A previous clinical study by our group showed that BoNT‐A injection may affect muscle growth in children with CP by altering protein expression, including thioredoxin, and signal pathways, including PI3K‐Akt [25]. Because skeletal muscle is the primary site of BoNT‐A, its proteomic profile may more directly reflect BoNT‐A related molecular changes, such as (e.g., synaptic vesicle protein degradation, neurogenerative signaling). However, muscle biopsy in children is constrained by ethical considerations. Therefore, investigating changes in protein expression in spastic muscle and the associated molecular mechanisms after BoNT‐A administration in animal models of CP is clinically important.

This study explores the mechanisms underlying nerve sprouting following BoNT‐A injection in a spastic hemiplegic cerebral palsy (HCP) rat model. Proteomic analysis of the gastrocnemius muscle revealed a temporal shift in differentially expressed protein (DEP) enrichment: from protein synthesis and vesicle transport (4 weeks) to metabolic regulation (12 weeks). A total of 25 shared DEPs were identified across the two time points, among which 11 exhibited expression dynamics consistent with the therapeutic efficacy of BoNT‐A. Further analysis identified Rtn1 and Sar1b as candidate regulators whose expression paralleled the dynamics of neural sprouting, providing a basis for their further investigation as potential molecular targets for prolonging the therapeutic effects of BoNT‐A.

## 2 Materials and Methods

### 2.1 Animals

The rat strain used in the experiment is Wistar rat. Ten specific pathogen‐free (SPF‐grade) pregnant Wistar rats were procured from Spover Biotechnology Co. Ltd. (License No. SCXK Yue 2020‐0055). The dams delivered 103 pups in total. All experimental animals were accommodated in a standard SPF‐grade environment maintained at 22°C–24°C with a 12‐h light/dark cycle and provided with ad libitum access to food and water. This study was performed in strict accordance with institutional animal care guidelines throughout the experiment.

### 2.2 Spastic HCP Rat Model Establishment

**FIGURE 1:**
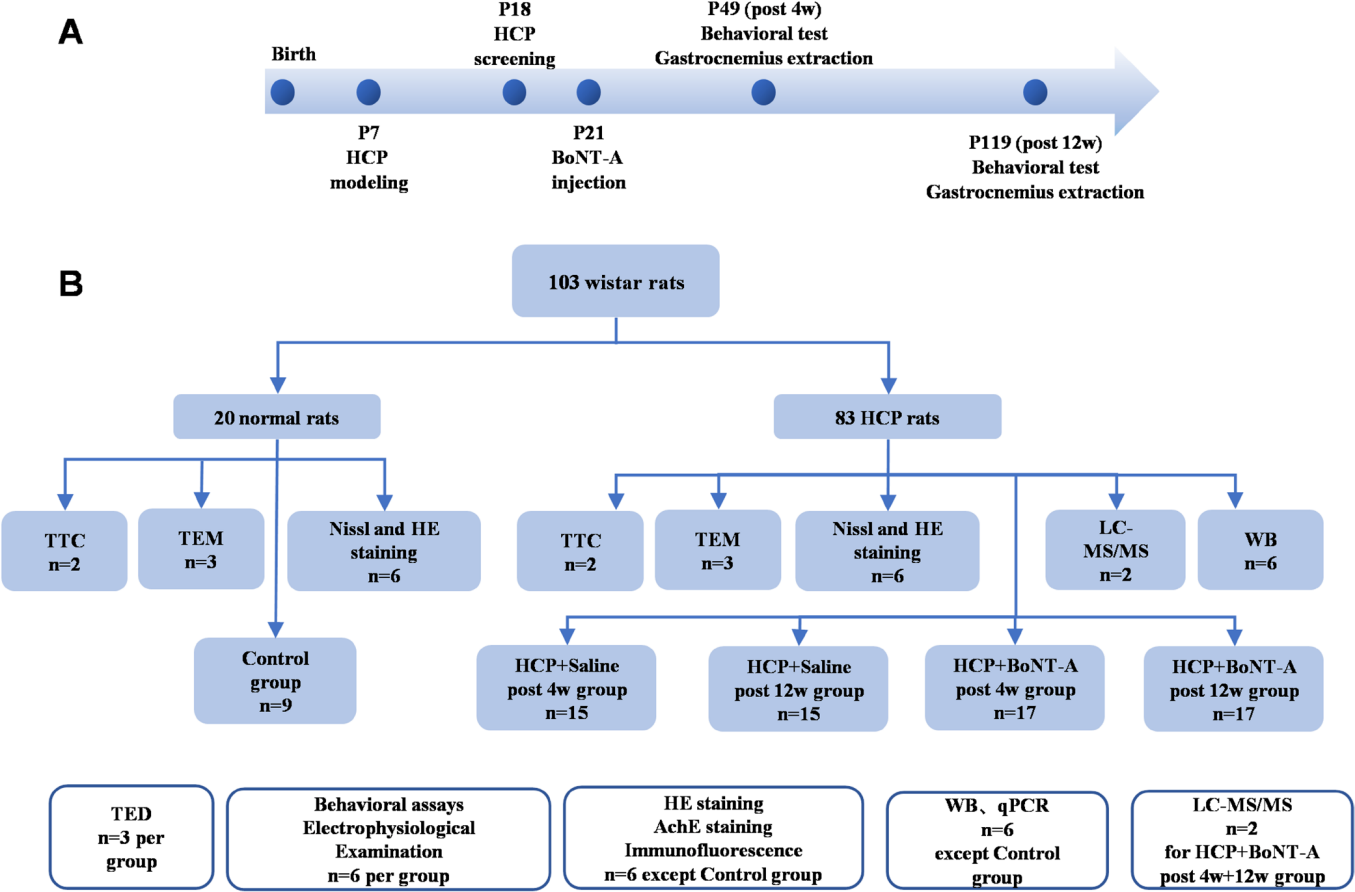
The timeline and rats assignment of the whole experiment. (A) Experimental timeline arrangement process. (B) Experimental arrangement. BoNT‐A, botulinum neurotoxin A; HCP, Hemiplegic cerebral palsy; HE, hematoxylin–eosin; LC–MS/MS, liquid chromatography–tandem mass spectrometry; TEM, transmission electron microscopy; TTC, triphenyltetrazolium chloride; WB, western blot.

The inhalation anesthetic drugs used in the experiment were all special anesthetic agents for animals—isoflurane. The modified Rice‐Vannucci method was used to establish a hypoxic–ischemic model as previously described [26]. On postnatal Day 7 (P7), the left common carotid artery of rats was ligated under anesthesia (3% isoflurane) to achieve ischemia, followed by 3 h of hypoxia (8% O_2_, 37°C). The experiment timeline and rat assignment of the whole experiment were shown in Figure 1. Successful ischemia was confirmed by triphenyltetrazolium chloride (TTC) staining at 24 h post‐operation. At P18, model success was further verified by a significant reduction in rotarod retention time and hindlimb grip strength, alongside pathological damage in the motor cortex (Nissl staining). If hematoxylin and eosin (HE) staining showed morphological changes of gastrocnemius muscle, and modified Ashworth scale (MAS) and H‐reflex of electrophysiological evaluation increased, it suggested that spastic HCP rat model was successfully established.

### 2.3 BoNT‐A Injection

At P21, the rats with a body weight of approximately 50 g (regardless of gender) were immobilized and anesthetized to remove the hair on the right leg, and muscles were exposed. Then, 50 U BoNT‐A (BOTOX, Allergan Inc., USA) was diluted to 0.01 U/μL in saline and injected into gastrocnemius medial and lateral of right leg based on body weight (5 U/kg), saline as placebo injection. All rats were grouped into five according to intervention and time: pre‐injection (HCP‐Pre), saline post‐injection 4 weeks (HCP + saline post 4w), saline post‐injection 12 weeks (HCP + saline post 12w), BoNT‐A post‐injection 4 weeks (HCP + BoNT‐A post 4w), BoNT‐A post‐injection 12 weeks (HCP + BoNT‐A post 12w). Muscle samples were collected at pre‐injection (P21), post‐injection 4 weeks and post‐injection 12 weeks.

### 2.4 Behavioral Test

Motor coordination, hindlimb grip strength, and muscle tone were assessed at pre‐injection, post‐injection 4 weeks, and 12 weeks (*n* = 18 per group, except muscle tone *n* = 6). Digit abduction score (DAS) was used to assess BoNT‐A induced paralysis at post‐injection 3 days (*n* = 6 per group) as previously described [27]. Rotarod test for assessing motor coordination, rats underwent pretraining (3 × 5 min at 30 rpm), followed by tests increasing from 30 to 40 rpm over 300 s. Latency to fall was recorded across three trials and averaged; hindlimb grip strength was measured with a digital grip tester (INNOTEG, Guangzhou) by placing the hindlimbs on the grip plate and pulling the tail backward; the force value was recorded and averaged over three trials; muscle spasticity was evaluated using the MAS via passive ankle dorsiflexion while the rat was in a relaxed, crawling position [28, 29].

### 2.5 Electrophysiological Examination

H‐reflex and compound muscle action potential (CMAP) were recorded using a multichannel recorder (CHENGYI, China) in anesthetized (1.5%–2% isoflurane) HCP + saline and HCP + BoNT‐A group (*n* = 6) at pre‐injection, post‐injection 4 and 12 weeks. Under prone‐fixed on a 37°C pad, tibial nerve was stimulated via popliteal fossa (anode distal, cathode proximal) from 0.5 mA (20s delay, 0.5 ms pulse, 5 Hz) until stable M‐waves emerged. H‐reflex and CMAP were recorded from interosseous muscles (4th–5th metatarsals) with sensitivity 1.0 mV/D, sweep speed 1 ms/D, bandpass 100–1000 Hz. Grounding wire was subcutaneously placed at the tail [30].

### 2.6 Histological Morphology

Gastrocnemius muscles were harvested from HCP + saline and HCP + BoNT‐A group (*n* = 6) at pre‐injection, post‐injection 4 and 12 weeks, then the tissue was weighed, fixed, dehydrated, and embedded. Transverse sections (5 μm paraffin, 10/50 μm frozen) were prepared from the mid‐belly region for histological analysis.

Hematoxylin and eosin (HE) staining. After the paraffin sections were deparaffinized and rehydrated, they were stained with hematoxylin and eosin (Solarbio, G1120), and the cross‐sectional area of muscles was observed and recorded under a microscope (Leica DM250, Germany) [31].

Acetylcholinesterase (AChE) staining. Ten‐micrometer frozen tissue sections were fixed in pre‐cooled 10% formaldehyde calcium for 10 min and subsequently rinsed with distilled water. The AChE incubation solution was prepared by appropriately mixing the reagents (Saint‐Bio, RN001A). Sections were incubated in pre‐heated solution at 37°C in the dark until light brown, rinsed, counterstained with hematoxylin, dehydrated, mounted, and imaged [32].

Immunofluorescence (IF). After the paraffin embedding was processed according to the standard procedure [33], sections were incubated overnight at 4°C with primary antibodies including GAP43 (1:200, 16,971, Proteintech), IGF‐1 (1:200, PAA050Ra01, Cloud‐Clone), and S100 (1:50, IR504, DAKO), followed by a 1.5‐h incubation with the corresponding secondary antibody IgG H&L (1:200, Alexa Fluor 488, ab150077, Abcam) at room temperature. The cell nuclei were stained with 4′,6‐diamidino‐2‐phenylindole (DAPI). After washing with phosphate buffered saline (PBS), the slides were observed and recorded using a fluorescence microscope.

NMJs staining. Fifty‐micron thick frozen tissue sections were rehydrated in PBS for 5 min, then incubated with CF 594 α‐bungarotoxin (1:500, 00007, Biotium) for 1 h in a dark, humid chamber. After three PBS washes, the cell nuclei were stained with DAPI and imaged under confocal microscopy (Nikon, Japan) [34]. All images were analyzed using Image J software (v1.46r).

### 2.7 Western Blot

Total protein was extracted from 20 mg gastrocnemius muscle (*n* = 6) using 200 μL of lysate (RIPA: PMSF: protein phosphatase inhibitor, 100: 1: 1) and protein concentrations were determined after centrifugation. Proteins were separated by sodium dodecyl sulfate‐polyacrylamide gel electrophoresis and transferred to a polyvinylidene fluoride membrane. After blocked for 1 h, incubate with primary antibodies including GAP43 (1:1000, 16971, Proteintech), S100 (1:1000, PAA050Ra01, Cloud‐Clone), Sar1b (1:1000, 22292, Proteintech), Rtn1 (1:1000, 06812, Invitrogen), HSP90 (1:2000, 60318, Proteintech) overnight at 4°C. A was followed by 2 h incubation with corresponding secondary antibodies at room temperature [35]. Signals were detected with chemiluminescence reagent (Merck Millipore, WBKLS0500) and analyzed with Image J software.

### 2.8 Enzyme‐Linked Immunosorbent Assay

The same method as WB was used to homogenize the gastrocnemius muscle tissue and obtain the supernatant by centrifugation. Using ELISA kit (Sangon, D721014‐0048) to detect the expression level of IGF‐1 [36].

### 2.9 Quantitative Real‐Time PCR

Total RNA was extracted from the right gastrocnemius muscle (*n* = 6) using TRIZOL reagent [37], and synthesized cDNA using the Color Reverse Transcription Kit (EZBioscience, A0010CGQ). Quantitative PCR was performed using SYBR Green reagents (EZBioscience, A0012‐R2) and primers (provided by Tianyi Biotechnology Co. Ltd.), with β‐actin gene as the endogenous control. The sequences were shown in Supplementary S1 [38].

### 2.10 Proteomics Experiments

Muscle protein (*n* = 3) was quantified by Bicinchoninic Acid Assay, digested, and analyzed via LC–MS/MS. Peptides (2 μg) were separated on a C18 column using a 120‐min acetonitrile gradient and analyzed on a Q‐Exactive mass spectrometer in data‐dependent acquisition mode. Raw data were processed in MaxQuant (v1.5.6.0) against UniProt_Mouse_2016_09 (forward/reverse decoys) using label‐free quantification (LFQ). DEPs were defined by |log_2_ (fold change)| > 0.58 for subsequent GO/KEGG analyses [39].

### 2.11 Statistical Analysis

Statistical analysis was performed using IBM SPSS 25.0 and GraphPad Prism 9. Results were presented as mean ± standard deviation, and *p <* 0.05 was considered statistically significant. Intergroup comparisons were conducted using the *t*‐test or analysis of variance (ANOVA) with appropriate post hoc tests (LSD or Bonferroni). Correlations were evaluated by Pearson analysis. Image J (v1.46r) was used for quantitative analysis of the pictures.

## 3 Result

### 3.1 Spastic HCP Rat Model Was Successfully Established

**FIGURE 2:**
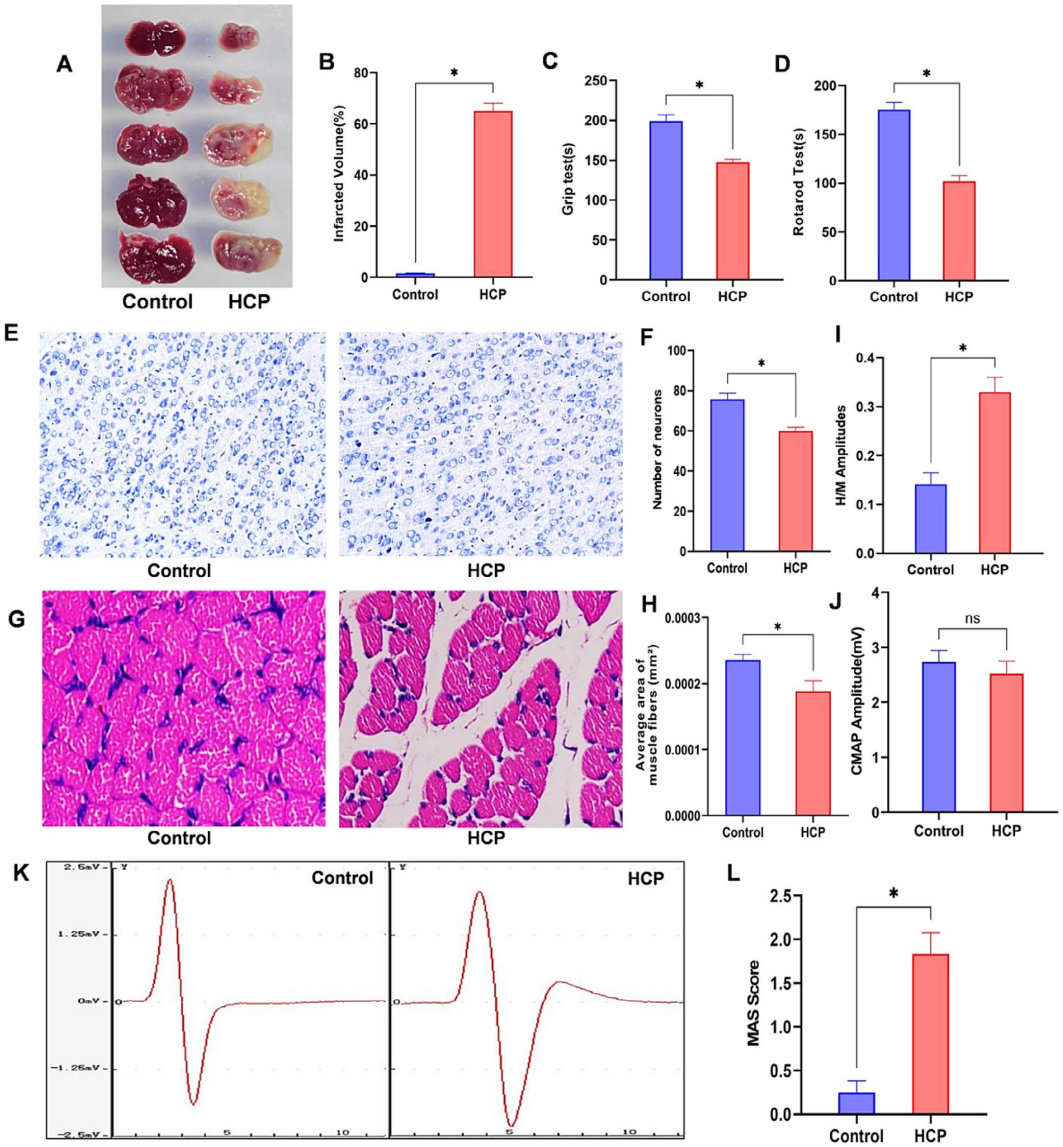
Spastic HCP model screening results. (A) Schematic diagram of ischemic infarction in rat brain with TTC staining 24 h after the HCP model was constructed. (B) Quantitative analysis of cerebral ischemic infarct size (*n* = 2). (C) Comparative analysis of hind limb grip in each group (*n* = 6). (D) Comparative analysis of the residence time of rats in each group (*n* = 6). (E) Schematic diagram of neurons after Nissl staining in each group of rats. (F) Comparison of the number of neurons in each group (*n* = 3). (G) Schematic diagram of HE staining of right gastrocnemius muscle in each group of rats. (H) Comparison of myofiber cross‐sectional area in each group (*n* = 6). (I) Comparative analysis of H/M ratio in each group (*n* = 6). (J) Comparative analysis of CMAP amplitude in each group (*n* = 6). (K) Schematic diagram of neuroelectrophysiological waveforms in each group L Comparative analysis of modified Ashworth scale scores in each group (*n* = 6). Data are means ± standard. *p < 0.05 indicated that there is a statistically significant difference between groups; ns indicates no statistical difference. CMAP, compound muscle action potential; HCP, hemiplegic cerebral palsy; MAS, Modified Ashworth scale; TTC, Triphenyltetrazolium chloride.

Compared with the control group, the HCP group exhibited prominent cerebral cortical infarctions (*p <* 0.05, Figure 2A,B), motor deficits (impaired hindlimb grip strength and rotarod latency) (*p <* 0.05, Figure 2C,D), and motor cortex neuronal loss (*p <* 0.05, Figure 2E,F). Pathological changes included the HCP group reduced gastrocnemius muscle fiber cross‐sectional area (*p <* 0.05, Figure 2G,H) and elevated H/M ratio (*p <* 0.05, Figure 2I,K), while CMAP showed no intergroup difference (*p* > 0.05, Figure 2J,K). Increased MAS scores in the HCP group confirmed spasticity (*p <* 0.05, Figure 2L), collectively verifying successful model establishment.

### 3.2 BoNT‐A Transiently Improves Behavioral Function and Induces Myofibers Atrophy and Ultrastructural Damage in Spastic HCP Rat

**FIGURE 3:**
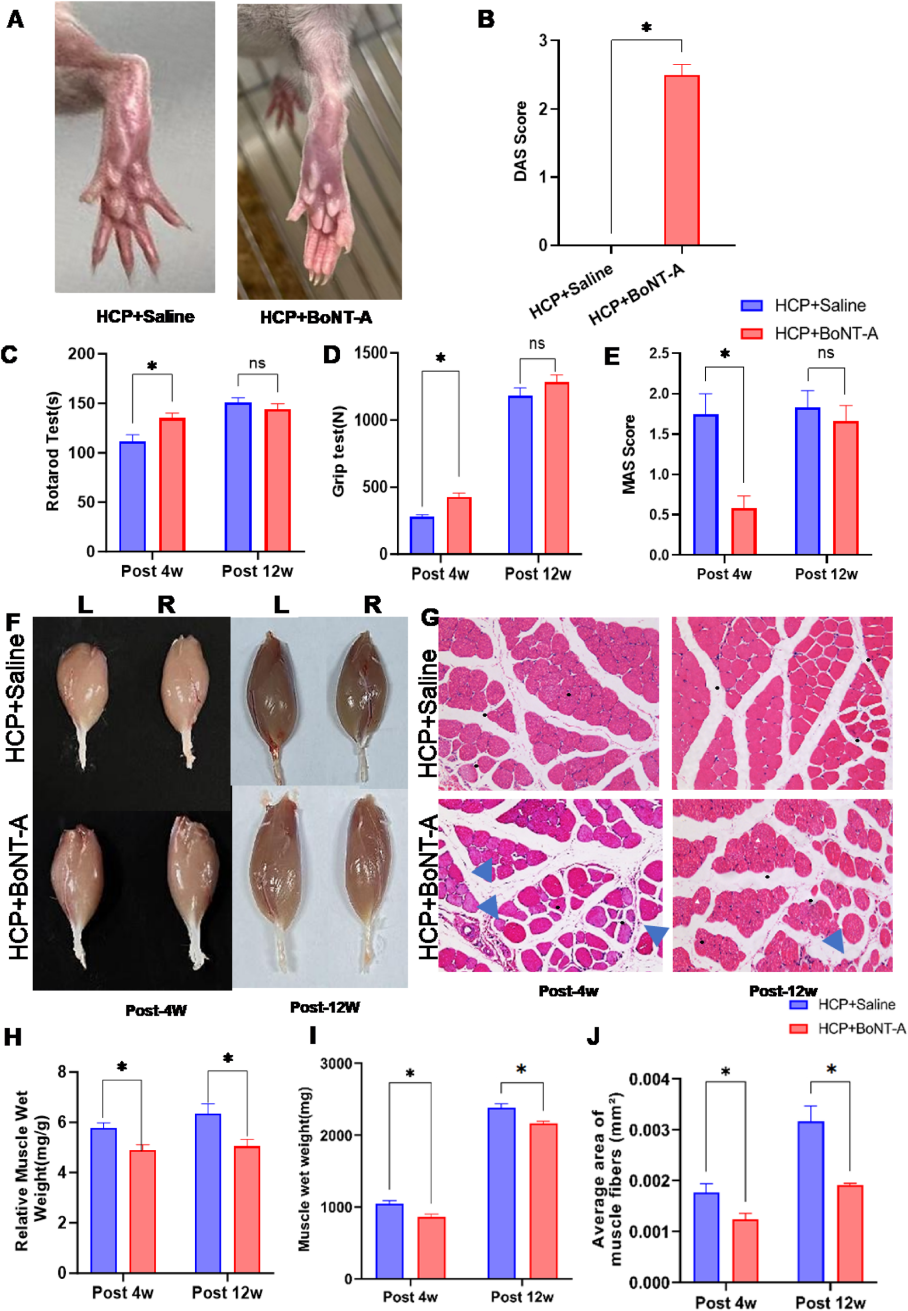
Behavioral function and changes in muscle morphology for spastic HCP rats with saline or BoNT‐A injection. (A) The hind limbs of the spastic HCP rats post‐injection 3 days. (B) Comparison of the DAS scores between different treatment groups (*n* = 6). (C) Comparison of the residence time in each group at the post‐injection 4 and 12 weeks (*n* = 6). (D) Comparison of grip size in each group at the post‐injection 4 and 12 weeks (*n* = 6). (E) Comparison of MAS in each group at the post‐injection 4 and 12 weeks (*n* = 6). (F) Representative image of the gastrocnemius muscle in each group at post‐injection 4 and 12 weeks. (G) Schematic diagram of HE staining in each group. (H–I) Comparing the relative muscle wet weight and muscle wet weight between the two groups at post‐injection 4 and 12 weeks (*n* = 6). (J) Comparing the average area of muscle fibers in each group at post‐injection 4 and 12 weeks. Data are means ± standard. *p < 0.05 indicated that there is a statistically significant difference between groups; ns indicates no statistical difference. HCP, hemiplegic cerebral palsy; L, represents the left side; MAS, Modified Ashworth scale; R, represents the right side; Post 4w, post‐injection 4 weeks; post 12w, Post‐injection 12 weeks.

Behaviorally, HCP + BoNT‐A group induced transient muscle paralysis (increased DAS score, *p <* 0.05, Figure 3A,B) in 3 days and significantly improved motor coordination, grip strength, and reduced muscle tone at 4 weeks compared to the HCP + saline group (*p <* 0.05, respectively, Figure 3C–E). However, all improvements in the HCP + BoNT‐A group returned to the same level as that of the HCP + saline group by 12 weeks, indicating a time‐limited therapeutic effect of BoNT‐A (*p* > 0.05, respectively, Figure 3C–E; Supplementary S2). Muscle morphology analysis revealed that BoNT‐A induced significant neurogenic atrophy (Figure 3F). At post‐injection 4 weeks, muscle fiber in the HCP + BoNT‐A group exhibited diminished fiber size, irregular contours, central nucleation, and collagen deposition. By post‐injection 12 weeks, pathological changes progressed to fiber size heterogeneity, structural disorganization, and persistent inflammation (Figure 3G). Compared with the HCP + saline group, the muscle wet weight and fiber cross‐sectional area in the HCP + BoNT‐A group were significantly reduced at both time points (*p <* 0.05, Figure 3H–J; Supplementary S3), confirming sustained BoNT‐A‐induced neurogenic atrophy.

### 3.3 Time‐Dependent Modulation of Neuromuscular Transmission and NMJs Structure After BoNT‐A Injection

**FIGURE 4:**
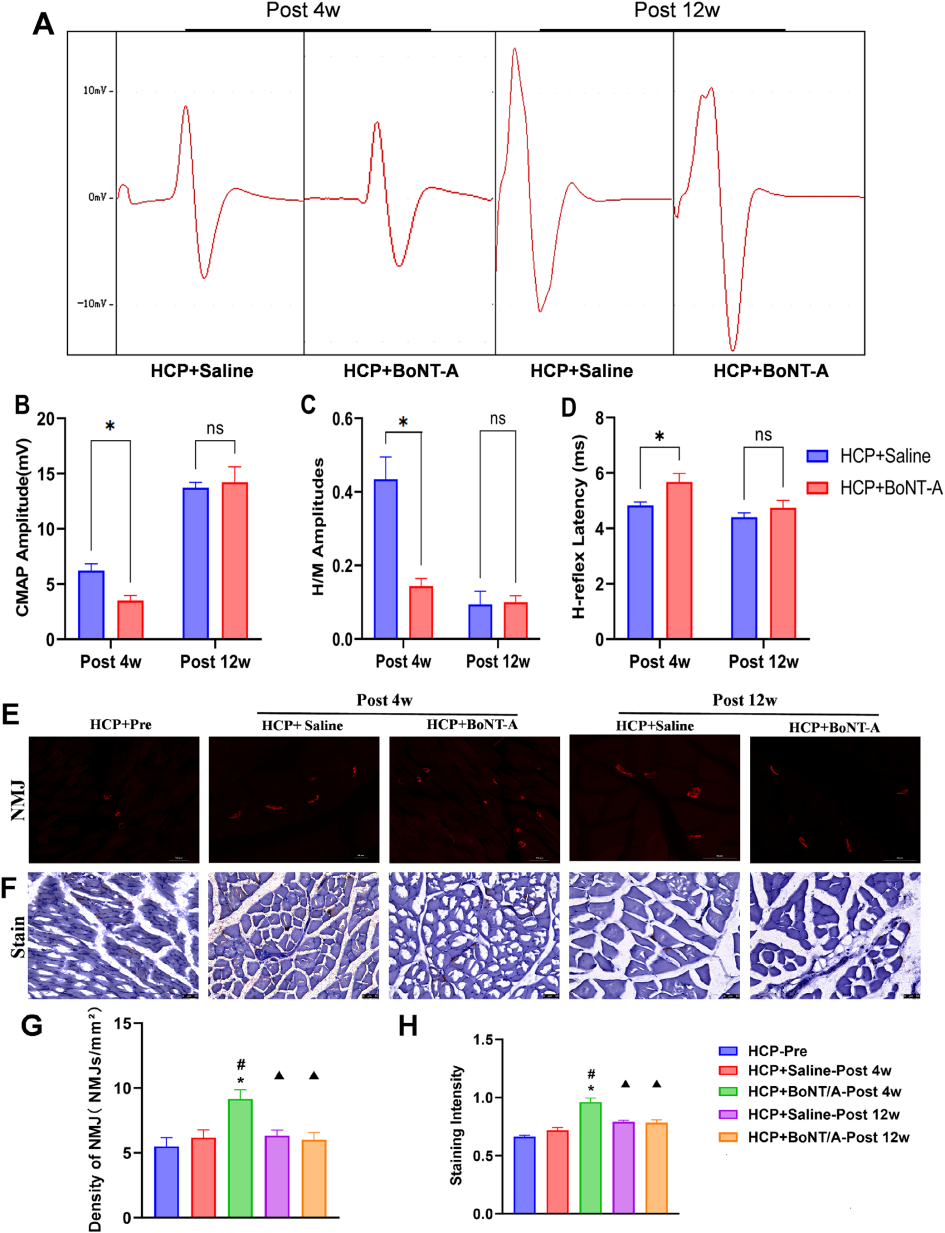
Neuroelectrophysiological and neuromuscular junctions (NMJs) structure changes in spastic HCP rats with saline or BoNT‐A injection. (A) Schematic diagram of neuroelectrophysiological waveforms at post‐injection 4 and 12 weeks. (B) Comparison of CMAP amplitude between the two groups at post‐injection 4 and 12 weeks (*n* = 6). (C) Comparison of H/M ratios between the two groups at post‐injection 4 and 12 weeks (*n* = 6). (D) Comparison of H‐wave amplitude between the two groups at post‐injection 4 and 12 weeks (*n* = 6). (E) Schematic diagram of fluorescent staining of NMJs. (F) Schematic diagram of acetylcholinesterase staining. (G) Comparison of NMJs density in rats in each group (*n* = 6). (H) Comparison of acetylcholinesterase staining in each group (*n* = 6). Data are means ± standard. ns indicates no statistical difference. *: Vs. HCP‐Pre group, *p* < 0.05; #: Vs. HCP + saline‐post 4w group, *p* < 0.05; ▲: Vs. HCP + BoNT‐A post 4w group, *p* < 0.05. HCP: Hemiplegic cerebral palsy; Post 4w: Post‐injection 4 weeks; Post 12w: Post‐injection 12 weeks.

Electrophysiological and morphological analyses revealed a time‐dependent effect of BoNT‐A. At 4 weeks post‐injection, the HCP + BoNT‐A group showed significantly reduced CMAP amplitude, H/M ratio, and increased latency period of H reflex (*p <* 0.05, respectively, Figure 4A–D). The two‐way analysis of variance revealed a significant interaction for both the density of NMJ (*F* (2, 30) = 4.461, *p <* 0.05) and the activity of acetylcholinesterase (*F* (2, 48) = 20.80, *p <* 0.001). Among them, the NMJ density (*p <* 0.05, respectively, Figure 4E,G) and AChE activity (*p <* 0.05, respectively, Figure 4F,H) in the HCP + BoNT‐A post 4w group were significantly higher than those in the HCP + saline post 4w group and the HCP + pre group, indicating peak neuro‐blockade and concomitant nerve sprouting.

### 3.4 Temporal Upregulation of Nerve Growth‐Related Proteins Post BoNT‐A Injection

**FIGURE 5:**
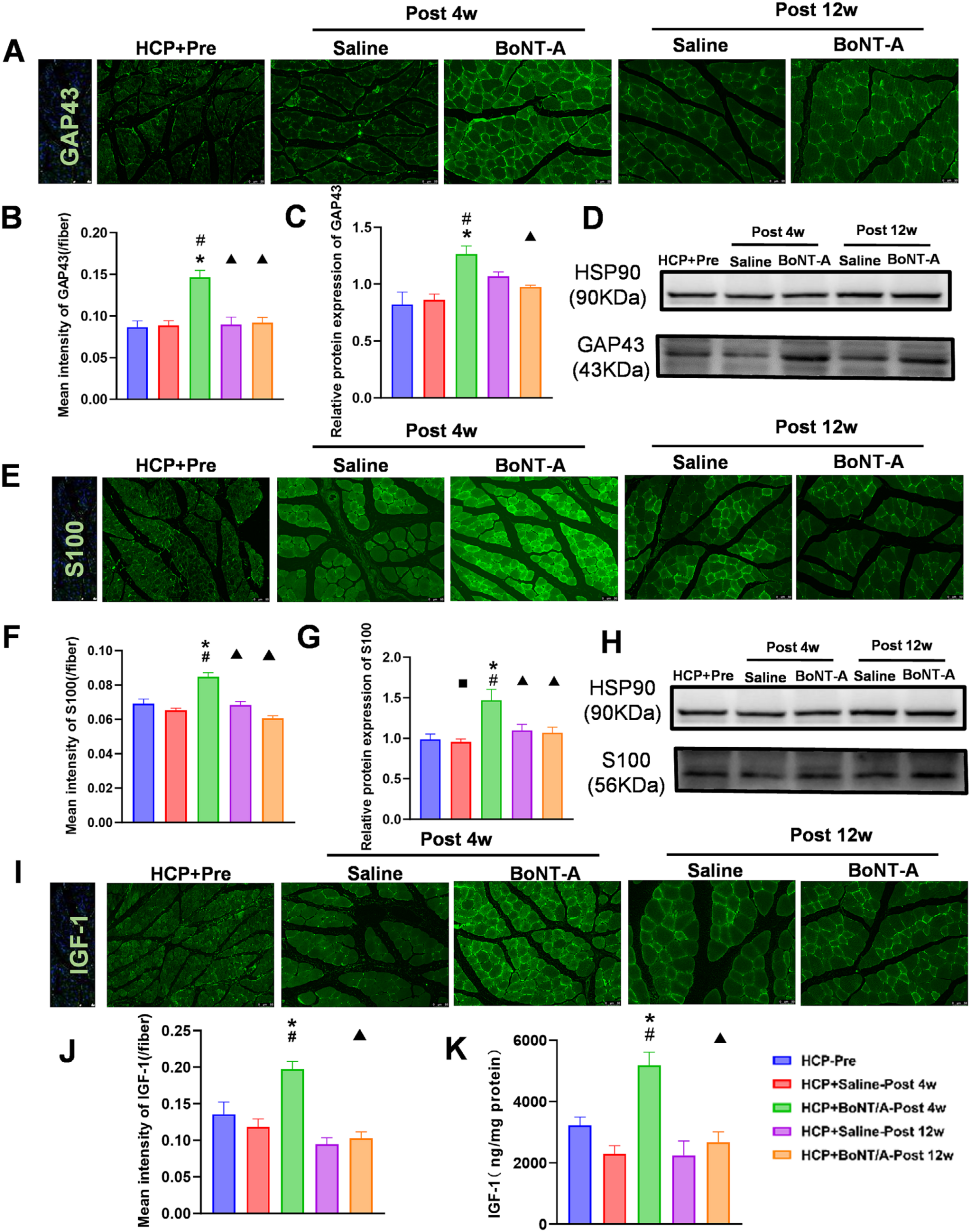
Expression of nerve growth‐related proteins in spastic HCP rats of different groups. (A) Expression of GAP43 in the gastrocnemius muscle as assessed by immunofluorescence staining. (B) Comparison of the fluorescence intensity of GAP43 in each group (*n* = 6). (C) Comparison of the relative protein expression of GAP43 in each group (*n* = 6). (D) Expression of GAP43 in the gastrocnemius muscle as assessed by western blot. (E) Expression of S100 in the gastrocnemius muscle as assessed by immunofluorescence staining. (F) Comparison of the fluorescence intensity of S100 in each group (*n* = 6). (G) Comparison of the relative protein expression of S100 in each group (*n* = 6). (H) Expression of S100 in the gastrocnemius muscle as assessed by western blot. (I) Expression of IGF‐1 in the gastrocnemius muscle as assessed by immunofluorescence staining. (J) Comparison of the fluorescence intensity of IGF‐1 in each group (*n* = 6). (K) Expression levels of IGF‐1 in the gastrocnemius muscle as assessed by ELISA. Data are means ± standard. *: Vs. HCP‐Pre group, *p* < 0.05; #: Vs. HCP + saline‐post 4w group, *p* < 0.05; ▲: Vs. HCP + BoNT‐A post 4w group, *p* < 0.05. BoNT‐A: HCP + BoNT‐A group; HCP, hemiplegic cerebral palsy; NMJs, neuromuscular junctions; Saline, HCP + saline group; Post 4w: Post‐injection 4 weeks; Post 12w: Post‐injection 12 weeks.

The two‐way variance analysis of neuroplasticity markers revealed significant group × time interactions for GAP43 (IF: *F* (2, 48) = 9.544, *p <* 0.001; WB: *F* (2, 30) = 6.333, *p <* 0.01), S100 (IF: *F* (2, 30) = 20.33, *p <* 0.001; WB: *F* (2, 36) = 7.316, *p <* 0.01), and IGF‐1 (IF: *F* (2, 48) = 5.981, *p <* 0.01; ELISA: *F* (2, 12) = 10.03, *p <* 0.01). The specific results can be found in Supplementary S4. Furthermore, WB, ELISA, and IF analyses confirmed that the expressions of the three neuroplasticity markers were significantly upregulated 4 weeks after BoNT‐A injection (*p <* 0.05, respectively, Figure 5). However, by 12 weeks, their expression returned to baseline levels, indicating a transient enhancement of neural regeneration potential following BoNT‐A treatment.

### 3.5 Correlation Between Motor Function, Muscle Morphology and NMJs Remodeling in Spastic HCP Rat and Label‐Free Quantification of Rat Proteomes

**FIGURE 6:**
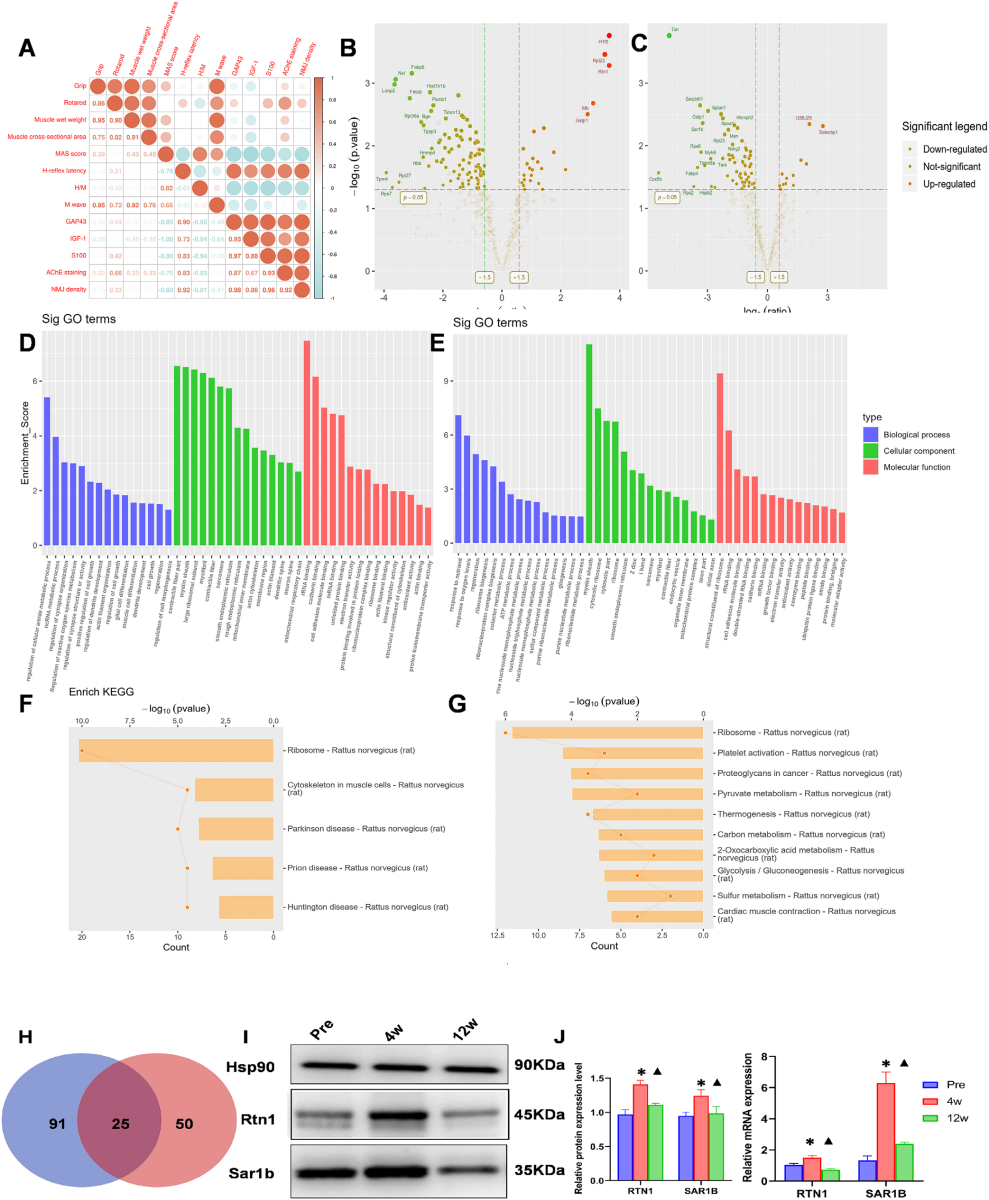
Results of correlation analysis, differential protein expression and functional verification in spastic HCP rats. (A) Correlation analysis of motor function, neuroelectrophysiology and related protein factors. The horizontal and vertical axes represent detection indices. Circles indicate correlation coefficients, with color and size reflecting correlation features: Red for positive, blue for negative. Color depth and circle size correspond to the absolute value of the Pearson correlation coefficient, where darker colors signify stronger correlations. (B, C) Differential protein volcano maps. *y*‐axis: −log10(*p*‐value); *x*‐axis: Log2(ratio). Points outside the two vertical lines and above the horizontal line denote significantly differentially expressed proteins, with green for downregulated, red for upregulated, and light‐yellow for non‐statistically different proteins (*n* = 2). Differentially expressed proteins were filtered with criteria of *p* < 0.05, FC > 1.5 or FC < 0.83. (D, E) Differential proteins Gene Ontology (GO) function annotation analysis, with (D) for Pre vs. 4w and (E) for 4w versus 12w. (F–G) Encyclopedia of Genes and Genomes (KEGG) pathway enrichment category of the differentially expressed proteins, with (F) for Pre versus 4w and (G) for 4w vs. 12w. (H) Venn diagram summarizing the number of differential and overlapping proteins in Pre, 4w and 12w. (I) Expression level of the Rtn1 and Sar1b by western blot (*n* = 6). (J) Statistical analysis of the grayscale values and mRNA for the expression levels of Rtn1, Sar1b, and the internal reference protein (*n* = 6). Data are means ± standard. *: vs. Pre group, *p* < 0.05; ▲: vs. post 4w group, *p* < 0.05. BoNT‐A, botulinum neurotoxin A; HCP, hemiplegic cerebral palsy; Pre, before BoNT‐A injection; Post 4w, post‐injection 4 weeks; Post 12w, post‐injection 12 weeks.

The Pearson correlation revealed significant associations among key parameters in Figure 6A. The motor function (grip strength and rotational rod performance) showed a strong positive correlation with muscle wet weight (*r* = 0.90–0.95, *p <* 0.05); spasticity severity (MAS) was significantly negatively correlated with the latency of H‐reflex (*r* = −0.78) and positively correlated with the H/M ratio (*r* = 0.82); the density of NMJs is strongly positively correlated with neural regeneration markers GAP43 (*r* = 0.98), S100 (*r* = 0.96), and IGF1 (r = 0.86); AChE activity is positively correlated with S100 expression (*r* = 0.93). However, the levels of AChE and NMJs density have no significant correlation with motor performance. Specific results of proteomic analysis are presented in Supplementary S5 and S6.

Proteomic analysis identified 116 DEPs at 4 weeks post‐BoNT‐A and 75 DEPs between 4‐ and 12‐week time points (Figure 6B,C). Functional enrichment linked these DEPs to amino acid metabolism, protein synthesis, signal transduction, oxidative stress, and immune responses. GO analysis showed that DEPs in post‐injection 4 weeks (HCP‐Pre vs. HCP + BoNT‐A post 4 weeks) focused on biological processes such as synaptogenesis, energy metabolism, cytoskeletal organization, and support for intracellular structures and neurons (Figure 6D). By 12 weeks, DEPs (HCP + BoNT‐A post 4 weeks vs. HCP + BoNT‐A post 12 weeks) shifted to ribosomal biogenesis and tissue regeneration, molecular function (Figure 6E). KEGG pathway analysis further indicated initial involvement in ribosome function, muscle cytoskeletal organization (Figure 6F), transitioning later to pathways of ribosome assembly, platelet activation, glycolysis/neogenesis, and myocardial contraction (Figure 6G).

Analysis identified 25 shared DEPs at 4 and 12 weeks (Figure 6H), among which 11 exhibited expression dynamics aligned with BoNT‐A efficacy. Reticulon‐1 (Rtn1) and Sar1b implicated in synaptic growth were prioritized. Their expression peaked at 4 weeks and declined by 12 weeks, as validated by WB and qPCR, suggesting key roles in BoNT‐A‐induced nerve sprouting (Figure 6I,J).

## 4 Discussion

This study clarifies the molecular transitions underlying the transient efficacy of BoNT‐A therapy. Our proteomic profiling reveals a distinct temporal shift in pathway enrichment: an early phase (4 weeks) dominated by protein synthesis and vesicular transport, followed by a late phase (12 weeks) characterized by metabolic reprogramming. This temporal progression was associated with compensatory nerve sprouting and restoration of synaptic function and contributed to the limited duration of BoNT‐A's therapeutic effect. Our findings support nerve sprouting as a potential contributor to the limited duration of BoNT‐A's therapeutic effects and characterize molecular changes associated with this process, thereby identifying candidate targets for further investigation.

A model that reliably reproduces muscle spasticity is essential for evaluating BoNT‐A therapy. Using the classic Rice‐Vannucci model at P7–8 (equivalent to human gestational weeks 35–40) [26], we established a spastic HCP rat model that recapitulated several key features, including cerebral infarction, neuronal loss, myelin damage, motor dysfunction, and histopathological changes in skeletal muscle [40, 41]. Spasticity was supported by increased MAS scores [42], shortened H‐reflex latency, and an elevated H/M ratio, findings consistent with spinal reflex hyperexcitability in spastic CP [43].

Nerve sprouting at NMJs has been identified in previous studies as a major factor limiting the duration of BoNT‐A efficacy. Consistent with this, at 4 weeks after injection, we observed increased NMJ density, a broader spatial distribution of NMJs, and increased AChE activity, findings consistent with synaptic remodeling and recovery of neuromuscular transmission [17]. KEGG pathway analysis further showed that DEPs at 4 weeks after injection were enriched in pathways related to protein synthesis, energy metabolism, endoplasmic reticulum organization, and vesicle‐mediated transport at post‐injection 4 weeks, with significant increases in proteins involved in translation (e.g., Rpl23, Rps18) and cytoskeletal remodeling (e.g., Bgn, Capzb). Integrating previous finding of increased proteins associated with synaptic remodeling, such as Agrin, IGF‐1R, and neural cell adhesion molecules, after BoNT‐A injection [16], these results suggest that the coordinated molecular changes observed at 4 weeks may represent a compensatory adaptive response. This response meets the heightened biosynthetic and energy demands required for synaptic vesicle transport and release, thereby facilitating structural remodeling of the NMJ and recovery of neuromuscular transmission through nerve sprouting.

By 12 after BoNT‐A injection, proteomic signatures had shifted towards pathways related to metabolic regulation, including glycolysis, pyruvate metabolism, and sulfur metabolism, alongside continued ribosomal biogenesis. This shift coincided with the decline in therapeutic efficacy and recurrence of muscle spasticity and may reflect restored neuromuscular transmission and increased energy demands [44]. Concurrently, sustained ribosomal biogenesis may support ongoing protein synthesis and repair mechanisms to maintain NMJ homeostasis. These molecular dynamics are consistent with the initial improvement observed at 4 weeks and the loss of therapeutic benefit by 12 weeks in our study, as well as with reported symptom recurrence 3–6 months after injection [45]. Furthermore, the metabolic profile is consistent with reported alterations in plasma metabolite pathways related to energy metabolism, including the tricarboxylic acid cycle, β‐alanine, and glycine‐serine–threonine metabolism [22]. Therefore, the increased abundance of energy metabolism‐related proteins may reflect both regained neuromuscular transmission and re‐established spasticity as the pharmacological effects of BoNT‐A diminish.

To further characterize the temporal proteomic changes, we integrated the gastrocnemius muscle proteomic datasets obtained before injection and at 4 and 12 weeks after injection. This analysis identified 116 DEPs between the pre‐injection and 4‐week assessments and 75 DEPs between the 4‐ and 12‐week assessments. Among these, 25 DEPs exhibited stable and persistent changes in expression across both time points, which suggests that this subset of proteins may play an indispensable and sustained regulatory role in the long‐term regulatory network underlying neuromuscular recovery and neural structural remodeling. Among these candidates, Rtn1 and Sar1b were prioritized because their expression increased at 4 weeks and subsequently decreased by 12 weeks.

Rtn1, known to maintain endoplasmic reticulum (ER) homeostasis and support secretory vesicle generation and axonal transport, may facilitate synaptic plasticity and nerve sprouting [46, 47]. Its structural similarity to the axonal growth inhibitor Rtn4 further suggests a regulatory role in neural branching [48, 49]. Sar1b, a key protein involved in COPII vesicle formation, mediates ER‐to‐Golgi transport of proteins and lipids [50]. In this study, Sar1b was significantly upregulated at 4 weeks, suggesting a potential role in modulating synaptic vesicle transport during nerve sprouting. This interpretation is consistent with previous evidence implicating Sar1b in axonal migration and morphogenesis in cortical neurons [51]. Based on the early changes in cytoskeletal protein expression after BoNT‐A injection, we propose that BoNT‐A triggers nerve sprouting partly by coordinating Rtn1‐ and Sar1b‐mediated vesicle generation, transport, and membrane remodeling [52]. Their coordinated involvement in ER‐Golgi function and vesicular transport likely underpins the structural dynamics required for sprouting and functional neuromuscular recovery. Future studies could further clarify the mechanism of Rtn1 and Sar1b in the nerve sprouting after injection by either inhibiting Rtn1 or overexpressing Sar1b.

Additionally, previous studies have shown that although BoNT‐A reduces muscle spasticity and improves motor function in CP, it may also result in reductions in muscle mass of 18%–81% and muscle cross‐sectional area reduction of 16%–56% [53]. However, at post‐injection 4 weeks, we found that Ca^2+^‐binding protein S100, which is involved in muscle repair and synaptic stability, was also significantly upregulated in DEPs, suggesting that the body may counteract the risk of atrophy following BoNT‐A injection through S100 [54, 55].

This study has several limitations. First, the functional roles and molecular mechanisms of the candidate proteins in BoNT‐A induced nerve sprouting were not validated in vivo. Second, muscle morphology was assessed only through 12 weeks after injection, potentially missing later changes and long‐term effects. Future research will focus on in vitro and in vivo functional validation of target proteins and elucidating their molecular mechanisms in regulating nerve sprouting. Additionally, the observation period will be extended to gain a more comprehensive understanding of the long‐term effects of BoNT‐A, ultimately aiming to provide strong theoretical support for optimizing clinical treatment strategies.

## 5 Conclusion

In summary, this study elucidates that BoNT‐A facilitates NMJ recovery through temporal regulation of vesicular transport, protein synthesis, and energy metabolism, yet this compensatory nerve sprouting ultimately limits its therapeutic duration. We identify Sar1b and Rtn1 as key regulators of this process, offering promising targets for developing strategies to prolong BoNT‐A's clinical efficacy.

## Author Contributions

Mengru Zhong and Huijuan Lin: visualization, methodology, conceptualization, software, formal analysis, writing‐original draft, writing‐review and editing. Xubo Yang, Ting Gao, and Liru Liu: methodology, conceptualization. Yunlan Xie and Tingting Peng: visualization, drafting and revising the article. Jie Luo: writing – grammar and polishing. Lu He and Hongmei Tang: ensuring accountability for the accuracy and integrity of the work. Kaishou Xu: conceptualization, funding acquisition, supervision, project administration, writing‐review and editing.

## Funding

This work was supported by Brain Science and Brain‐like Intelligence Technology—National Science and Technology Major Project (2021ZD0200500), National Natural Science Foundation of China (82472598) and Natural Science Foundation of Guangdong Province (2025A1515010379).

## Ethics Statement

This study was approved by the Laboratory Animal Committee of Guangzhou Women's and Children's Medical Center (ethical approval no. RSDW‐2023‐01409).

## Conflicts of Interest

The authors declare no conflicts of interest.

## Supporting information


**Supplementary S1**. Primer sequences used for qRT‐PCR.
**Supplementary S2**. The results of hindlimb grip and rotarod test among groups.
**Supplementary S3**. Statistical analysis of the results of wet weight and wet weight/body weight of right gastrocnemius muscle in each group.
**Supplementary S4**. The results of the two‐factor analysis of variance.
**Supplementary S5**. List of Significantly Regulated Protein between Different Groups.
**Supplementary S6**. Changes of Protein Level between Different Groups.

## Data Availability

The datasets generated and/or analyzed during the current study are available from the corresponding author on reasonable request. For any specific inquiries regarding the data or materials used in this study, please contact the corresponding author.
