# Supplementary material for: Temporal Neuromuscular Adaptations and Proteomic Signatures Following Botulinum Neurotoxin A Injection in Spastic Hemiplegia Rats
Source: CNS Neurosci Ther. 2026 Jul 28;32(7):e71057. doi: 10.1002/cns.71057 (PMC13410740; doi:10.1002/cns.71057)
Supplement: Supplementary file 1 — Supplementary S1. Primer sequences used for qRT‐PCR. Supplementary S2. The results of hindlimb grip and rotarod test among groups. Supplementary S3. Statistical analysis of the results of wet weight and wet weight/body weight of right gastrocnemius muscle in each group. Supplementary S4. The results of the two‐factor analysis of variance. Supplementary S5. List of Significantly Regulated Protein between Different Groups. Supplementary S6. Changes of Protein Level between Different Groups. [file CNS-32-e71057-s001.doc]

**Temporal neuromuscular adaptations and proteomic signatures following botulinum neurotoxin A injection in spastic hemiplegia rats**

Mengru Zhong^1†^, Huijuan Lin^1,2†^, [Xubo Yang](https://pubmed.ncbi.nlm.nih.gov/?term=Yang+X&cauthor_id=38456375)^1^, [Liru Liu](https://pubmed.ncbi.nlm.nih.gov/?term=Liu+L&cauthor_id=38456375)^1^, Yunlan Xie^1^, Tingting Peng^1^, Jie Luo^1^, Lu He^1^, Ting Gao^1^,Hongmei Tang^1^, Kaishou Xu^1^**^*^**, PhD

^1^ Department of Rehabilitation, Guangzhou Women and Children’s Medical Center, Guangzhou Medical University, Guangzhou, 510120, China

^2^ Department of Sports and Health, Guangzhou Sport University, Guangzhou 510500 Guangzhou, China

† Co-first authors

*** Corresponding authors:**

Kaishou Xu, MD, PhD

Department of Rehabilitation, Guangzhou Women and Children's Medical Center, Guangzhou Medical University, Guangzhou, 510120, People's Republic of China

Tel: +8613422250582(KSX)

E-mail: xksyi@126.com (KSX)

ORCID: 0000-0002-0639-3488(KSX)

**Supplementary material**

**Supplementary 1.** Primer sequences used for qRT-PCR

| Gene Names | Primer Sequences |
| --- | --- |
| SAR1B | Forward-ttcagaggagctcactattgctg  Reverse-tggtctgcacaatccacaaga |
| RTN1 | Forward-gaagaggaagctgtgagttcca  Reverse-ccgccagtacagaaggtcaat |
| β-Actin | Forward-AGCCATGTACGTAGCCATCCA  Reverse-TCTCCGGAGTCCATCACAATG |

**Supplementary 2.** The results of hindlimb grip and rotarod test among groups

| Groups | Rotarod test time (s) | Hindlimb grip strength(g) |
| --- | --- | --- |
| HCP+Pre | 94.62 ± 11.64 | 142.0 ± 10.38 |
| Control-Post 4w | 156 ± 36.54 | 518.8 ± 97.50 |
| HCP+Saline-Post 4w | 111.3 ± 24.13 | 278.7 ± 55.37 |
| HCP+BoNT-A-Post 4w | 135.3 ± 16.89 | 426.8 ± 40.3 |
| Control-Post 12w | 202.9 ± 56.86 | 1689 ± 218.6 |
| HCP+Saline-Post 12w | 144.1 ± 16.23 | 1181.4 ± 205.1 |
| HCP+BoNT-A-Post 12w | 77.57 ± 19.28 | 1282.3 ± 189.0 |

**Supplementary 3.** Statistical analysis of the results of wet weight and wet weight/body weight of right gastrocnemius muscle in each group

| Group | Wet muscle weight（mg） | | Muscle wet weight /body weight（mg/g) |
| --- | --- | --- | --- |
| Control+Pre | | 95.3 ± 16.3 | 2.38 ± 0.51 |
| HCP+Pre | | 85.3 ± 18.7 | 2.25 ± 0.42 |
| Control-Post 4w | | 1221.0 ± 105.9 | 6.03 ± 0.52 |
| HCP+Saline-Post 4w | | 995.8 ± 170.0 | 5.78 ± 0.20 |
| HCP+BoNT-A-Post 4w | | 874.1 ± 116.8 | 4.90 ± 0.20 |
| Control-Post 12w | | 2492.0 ± 100.9 | 6.69 ± 0.62 |
| HCP+Saline-Post 12w | | 2380.3 ± 130.5 | 6.36 ± 0.38 |
| HCP+BoNT-A-Post 12w | | 2161.4 ± 70.2 | 5.05 ± 0.28 |

**Supplementary 4**. The results of the two-factor analysis of variance

| Table1. Two-factor analysis of variance for the density of NMJs | | | | | |
| --- | --- | --- | --- | --- | --- |
| ANOVA table | SS | dF | MS | F (df_n_, df_d_) | P value |
| Interaction | 20.22 | 2 | 10.11 | F (2, 30) = 4.461 | P<0.05 |
| Type of injection | 29.56 | 2 | 14.78 | F (2, 30) = 6.520 | P<0.05 |
| Time | 7.111 | 1 | 7.111 | F (1, 30) = 3.137 | P>0.05 |

| Table2. Two-factor analysis of variance for the activity of AChE | | | | | |
| --- | --- | --- | --- | --- | --- |
| ANOVA table | SS | dF | MS | F (df_n_, df_d_) | P value |
| Interaction | 0.1842 | 2 | 0.09212 | F (2, 48) = 20.80 | P<0.001 |
| Type of injection | 0.2951 | 2 | 0.1475 | F (2, 48) = 33.31 | P<0.001 |
| Time | 0.08498 | 1 | 0.08498 | F (1, 48) = 19.19 | P<0.001 |

| Table3. Two-factor analysis of variance for the fluorescence intensity of GAP43 | | | | | |
| --- | --- | --- | --- | --- | --- |
| ANOVA table | SS | dF | MS | F (df_n_, df_d_) | P value |
| Interaction | 0.009705 | 2 | 0.004853 | F (2, 48) = 9.544 | P<0.001 |
| Type of injection | 0.01012 | 2 | 0.005059 | F (2, 48) = 9.950 | P<0.001 |
| Time | 0.005429 | 1 | 0.005429 | F (1, 48) = 10.68 | P<0.01 |

| Table4. Two-factor analysis of variance for the WB of GAP43 | | | | | |
| --- | --- | --- | --- | --- | --- |
| ANOVA table | SS | dF | MS | F (df_n_, df_d_) | P value |
| Interaction | 0.4172 | 2 | 0.2086 | F (2, 30) = 6.333 | P<0.01 |
| Type of injection | 0.4025 | 2 | 0.2013 | F (2, 30) = 6.111 | P<0.01 |
| Time | 0.09444 | 1 | 0.09444 | F (1, 30) = 2.868 | P=0.1007 |

| Table5. Two-factor analysis of variance for the fluorescence intensity of S100 | | | | | |
| --- | --- | --- | --- | --- | --- |
| ANOVA table | SS | dF | MS | F (df_n_, df_d_) | P value |
| Interaction | 0.001169 | 2 | 0.0005843 | F (2, 30) = 20.33 | P<0.001 |
| Type of injection | 0.0006721 | 2 | 0.0003361 | F (2, 30) = 11.69 | P<0.001 |
| Time | 0.0001372 | 1 | 0.0001372 | F (1, 30) = 4.771 | P<0.05 |

| Table6. Two-factor analysis of variance for the WB of S100 | | | | | |
| --- | --- | --- | --- | --- | --- |
| ANOVA table | SS | dF | MS | F(df_n_, df_d_) | P value |
| Interaction | 0.6527 | 2 | 0.3264 | F (2, 36) = 7.316 | P<0.01 |
| Type of injection | 0.3503 | 2 | 0.1751 | F (2, 36) = 3.926 | P<0.05 |
| Time | 0.2730 | 1 | 0.2730 | F (1, 36) = 6.120 | P<0.05 |

| Table7. Two-factor analysis of variance for the fluorescence intensity of IGF1 | | | | | |
| --- | --- | --- | --- | --- | --- |
| ANOVA table | SS | dF | MS | F (df_n_, df_d_) | P value |
| Interaction | 0.01724 | 2 | 0.008621 | F (2, 48) = 5.981 | P<0.01 |
| Type of injection | 0.03243 | 2 | 0.01622 | F (2, 48) = 11.25 | P<0.001 |
| Time | 0.01159 | 1 | 0.01159 | F (1, 48) = 8.044 | P<0.01 |

| Table8. Two-factor analysis of variance for the ELISA of S100 | | | | | |
| --- | --- | --- | --- | --- | --- |
| ANOVA table | SS | dF | MS | F(df_n_, df_d_) | P value |
| Interaction | 7329695 | 2 | 3664847 | F (2, 12) = 10.03 | P<0.01 |
| Type of injection | 4962040 | 2 | 2481020 | F (2, 12) = 6.788 | P<0.05 |
| Time | 5471639 | 1 | 5471639 | F (1, 12) = 14.97 | P<0.01 |

**Supplementary 5**. List of Significantly Regulated Protein between Different Groups

| Group name | Upregulated proteins | Downregulated proteins |
| --- | --- | --- |
| 4w vs Pre | 25 | 91 |
| 12w vs 4w | 12 | 63 |

**Supplementary 6**. Changes of Protein Level between Different Groups

| Num. | Protein name | 4w vs Pre | | 12w vs 4w | |
| --- | --- | --- | --- | --- | --- |
|  |  | Fold change | *P* value | Fold change | *P* value |
| 1 | Rps5 | 0.56^a^ | **0.01** | 0.44^a^ | **0.04** |
| 2 | Fabp4 | 0.3^a^ | **0.04** | 0.09^a^ | **0.02** |
| 3 | Rps18 | 0.61^a^ | **0.02** | 0.59^a^ | **0.04** |
| 4 | Rpl30 | 0.64^a^ | **0.01** | 0.62^a^ | **0.04** |
| 5 | Selenbp1 | 0.35^a^ | **0.01** | 6.84^b^ | **＜0.01** |
| 6 | Rtn1 | 12.49^b^ | **<0.01** | 0.32^a^ | **0.02** |
| 7 | Rps16 | 0.6^a^ | **0.02** | 0.42^a^ | **0.02** |
| 8 | Rplp2 | 0.55^a^ | **0.01** | 0.56^a^ | **0.02** |
| 9 | Gstp1 | 7.52^b^ | **＜0.01** | 0.13^a^ | **＜0.01** |
| 10 | Hp | 2.32^b^ | **0.02** | 0.46^a^ | **0.03** |
| 11 | Tst | 2^b^ | **0.03** | 0.53^a^ | **0.04** |
| 12 | Timm13 | 0.27^a^ | **<0.01** | 3.87^b^ | **0.02** |
| 13 | Ywhaq | 0.48^a^ | **0.02** | 0.41^a^ | **0.01** |
| 14 | Sar1b | 2.07^b^ | **0.02** | 0.11^a^ | **＜0.01** |
| 15 | Canx | 0.66^a^ | **0.02** | 0.46^a^ | **0.02** |
| 16 | Rpl23 | 11.26^b^ | **＜0.01** | 0.23^a^ | **0.01** |
| 17 | Serpinh1 | 0.46^a^ | **0.02** | 0.1^a^ | **＜0.01** |
| 18 | Pdia3 | 0.55^a^ | **0.02** | 0.46^a^ | **0.01** |
| 19 | Tars | 4.47^b^ | **0.02** | 0.21^a^ | **0.02** |
| 20 | Aldh2 | 1.66^b^ | **0.04** | 1.52^b^ | **0.04** |
| 21 | Hnrnpa2b1 | 0.57^a^ | **0.02** | 0.47^a^ | **0.01** |
| 22 | Uqcrq | 1.69^b^ | **0.03** | 0.34^a^ | **0.04** |
| 23 | Hist1h1b | 0.18^a^ | **＜0.01** | 0.43^a^ | **0.04** |
| 24 | Gpt | 1.72^b^ | **0.05** | 1.66^b^ | **0.03** |
| 25 | Vim | 0.57^a^ | **0.01** | 0.55^a^ | **0.01** |
| 26 | Apoa1 | 0.84^b^ | 0.91 | 0.21^a^ | **＜0.01** |
| 27 | Got1 | 1.45^b^ | 0.07 | 1.73^b^ | **0.04** |
| 28 | Ldha | 1.24^b^ | 0.47 | 1.88^b^ | **0.04** |
| 29 | Bgn | 0.19^a^ | **＜0.01** | 1.12^b^ | 0.8 |
| 30 | Dlat | 0.76^a^ | 0.15 | 1.59^b^ | 0.04 |
| 31 | Pkm | 1.51^b^ | 0.16 | 1.94^b^ | 0.03 |
| 32 | Sncg | 1.73^b^ | 0.04 | 0.37^a^ | **＜0.01** |

Pre: Before BoNT-A injection; 4w: The 4th week after BoNT-A injection; 12w: The 12th week after BoNT-A injection; Significant changes are marked in bold (P value < 0.05); a: Proteins with significant downregulation (fold change＜0.83); b: Proteins with significant upregulation (fold change＞1.5)
